# Women’s experience of transfer from midwifery unit to hospital obstetric unit during labour: a qualitative interview study

**DOI:** 10.1186/1471-2393-12-129

**Published:** 2012-11-15

**Authors:** Rachel E Rowe, Jennifer J Kurinczuk, Louise Locock, Ray Fitzpatrick

**Affiliations:** 1 National Perinatal Epidemiology Unit, Department of Public Health, University of Oxford, Old Road Campus, Oxford, OX3 7LF, United Kingdom; 2 Health Experiences Research Group, Department of Primary Care Health Sciences, University of Oxford, 23-38 Hythe Bridge Street, Oxford, OX1 2ET, United Kingdom; 3 Department of Public Health, University of Oxford, Old Road Campus, Oxford, OX3 7LF, United Kingdom

**Keywords:** Midwifery units, Birth centres, Intrapartum care, Transfer, Qualitative research

## Abstract

**Background:**

Midwifery units offer care to women with straightforward pregnancies, but unforeseen complications can arise during labour or soon after birth, necessitating transfer to a hospital obstetric unit. In England, 21% of women planning birth in freestanding midwifery units are transferred; in alongside units, the transfer rate is 26%. There is little high quality contemporary evidence on women’s experience of transfer.

**Methods:**

We carried out a qualitative interview study, using semi-structured interviews, with women who had been transferred from a midwifery unit (freestanding or alongside) in England up to 12 months prior to interview. Maximum variation sampling was used. Interviews with 30 women took place between March 2009 and March 2010. Thematic analysis using constant comparison and exploration of deviant cases was carried out.

**Results:**

Most women hoped for or expected a natural birth and did not expect to be transferred. Transfer was disappointing for many; sensitive and supportive care and preparation for the need for transfer helped women adjust to their changing circumstances. A small number of women, often in the context of prolonged labour, described transfer as a relief. For women transferred from freestanding units, the ambulance journey was a “limbo” period. Women wondered, worried or were fearful about what was to come and could be passive participants who felt like they were being “transported” rather than cared for. For many this was a direct contrast with the care they experienced in the midwifery unit. After transfer, most women appreciated the opportunity to talk about their experience to make sense of what happened and help them plan for future pregnancies, but did not necessarily seek this out if it was not offered.

**Conclusions:**

Transfer affects a significant minority of women planning birth in midwifery units and is therefore a concern for women and midwives. Transfer is not expected by women, but sensitive care and preparation can help women adjust to changing circumstances. Particular sensitivity around decision-making may be required by midwives caring for women during prolonged labour. Some apparently straightforward changes to practice have the potential to make an important difference to women’s experience of ambulance transfer.

## Background

Evidence from the Birthplace in England prospective cohort study supports offering healthy women with low risk pregnancies a choice about where to have their baby [1]. Depending upon where the woman lives, this choice may include planning birth in a midwifery unit. Midwifery units provide midwife-led care for women who are at low risk of complications at the start of labour and may be located on the same site as an obstetric unit (alongside) or at a separate location (freestanding) either in a hospital without obstetric services or in a building separate from any hospital [2]. In the year to 31^st^ March 2007, the most recent year for which these data are available, around 5% of women giving birth in England did so in a midwifery unit [3]. With the number of midwifery units increasing since 2007 [4] we might expect that figure to increase.

Midwifery units offer care to women who would be expected to have an uncomplicated labour and birth, but unforeseen complications can arise. When women need, or request, obstetric or anaesthetic care during labour or after the birth, or when babies need neonatal care, they are transferred to an obstetric unit, typically by wheelchair or trolley if the obstetric unit is on the same site or by car or ambulance if not. Transfer may take from a few minutes to substantially longer, depending upon the location of the midwifery unit and clinical urgency [5]. Overall, 21% of women planning birth in a freestanding midwifery unit are transferred, while in alongside units, the transfer rate is 26% [6]. These overall figures conceal variation between units of the same type and differences between groups of women with different characteristics. For example, transfer rates for women having their first baby are 36% in freestanding units and 45% in alongside units, while rates for multiparous women are much lower (9% and 13% respectively) [6]. Transfer is therefore an important consideration for women who are thinking about planning birth in a midwifery unit, for midwives working in midwifery units and for those planning and managing maternity services.

Within maternity care in general, but particularly within midwifery, a woman’s experience of labour and birth is seen explicitly as an important outcome, “not as an isolated clinical episode, but as a transformative life experience, enhancing the long term physical and emotional wellbeing of women and their families” [7]. There have been a number of evaluations of midwifery units, both in the UK and other parts of the world [8], some of which have explored women’s experience of being cared for within this type of unit [9-15]. A small number of mostly qualitative studies have explored women’s experience of transfer [15-20]. These studies give some indication of common themes, including feelings of disappointment after transfer [15,18-20], the importance of choice, control and continuity [15,18,19], and, from one study, a suggestion that women’s feelings of disappointment could be ameliorated by preparation and explanation during the antenatal period, during labour and postnatally [18]. However, these studies were carried out in the 1980s and 1990s and were all based on small convenience samples. Several also combined the experience of women ‘transferred’ from midwifery-led care during pregnancy or during labour from home with the experiences of women transferred during labour from a midwifery unit. There is therefore little high quality contemporary evidence on women’s experience of transfer at a time when the number of women planning to have their baby in a midwifery unit is likely to be increasing.

We carried out a qualitative study, as an adjunct study to the Birthplace in England Research Programme [21], to explore and describe the experiences, information and support needs of women transferred during labour or immediately after the birth from midwifery units in England. This paper reports on women’s hopes and expectations for birth, their experience of care in the midwifery unit and during transfer and their needs in the immediate postnatal period.

## Methods

The study was a qualitative interview study with women aged over 18 who had been transferred during labour or immediately after birth from a midwifery unit (freestanding or alongside) in England up to 12 months prior to interview.

### Ethical approval

The study was reviewed and approved by the National Research Ethics Service Berkshire Research Ethics Committee in March 2009 (Ref 08/H0505/208).

### Recruitment and sampling

Recruitment to the study took place between May 2009 and March 2010. Postnatal and research midwives in hospitals and midwives working in the community in three NHS hospital trusts in England gave information about the study to eligible women, who were invited to express an interest in taking part in the study by sending their contact details to the researcher (RER). Information about the study was also disseminated using NCT (National Childbirth Trust) email networks, notices posted on the online parent forum http://www.mumsnet.com, the website and Facebook page of the Birth Trauma Association and through an advertisement in a free daily metropolitan newspaper. In total, over the period of recruitment to the study, 95 women expressed an interest in taking part in the study. From these, purposive sampling was used to select 30 women, with the aim of achieving a sample with wide variation in women’s experience and the factors that may impact on experience, including ethnicity, age, socio-demographic status, marital status, geographical location, parity, type of midwifery unit, reason for transfer, mode of delivery and time since transfer. In practice, in the early stages of recruitment most women who expressed an interest in the study were invited to take part. As recruitment continued, in order to increase variation in the sample, women with particular characteristics, e.g. multiparous women, or those who had a particular transfer experience, e.g. emergency transfer, were invited to take part.

### Interviews

After reading the study information leaflet, women gave informed consent to take part in the study immediately prior to interview. One pilot interview was carried out, the data from which were included with subsequent interviews for analysis. Interviews began with a narrative stage in which the participant was invited to ‘tell the story’ of their transfer without interruption, followed by a semi-structured stage with follow-up questions and prompts about specific topics [22,23]. Topics covered included: reasons for planning birth in a midwifery unit; hopes and expectations for birth; expectations of transfer; feelings at the time and looking back; information and support needs; explanation and communication by staff; perceptions of care and longer term impact of the experience (Table 1). All interviews took place in participants’ homes and were audio-recorded, transcribed verbatim and imported into the qualitative data analysis software package NVivo 8 along with socio-demographic information for each participant [24].

**Table 1:** Interview topic guide

Introduce topic: experience of transfer from birth centre/midwifery unit where you planned to give birth to the hospital unit where you had your baby. Invite to tell story in as much detail as possible.	
Extra questions to follow up:	
Antenatal:	Reasons for deciding to have your baby in the midwifery unit / birth centre?
	Information given about the possibility of transfer? Written/verbal? Was it what you needed?
	Knowledge beforehand about why might be transferred and process?
	Anything you wish you’d known beforehand?
During labour (before transfer):	First inklings of complications / need for transfer: when, why, midwife communication, feelings?
	Understanding about reasons for transfer? Risks/benefits? Choice? Feelings?
	Transfer process? Knowledge, explanations, midwife accompany?
	Expectations of obstetric unit?
	Anything you would have liked but didn’t have?
	Waiting? Ambulance staff? Husband / partner accompanying? Feelings?
During transfer:	Describe journey? Midwife? Ambulance staff? Husband?
Arrival at hospital / delivery suite:	Communication / handover? Midwife continues to look after you?
	Hospital vs. midwifery unit? How did it feel?
	Husband / birth partner?
After the birth:	Explanation afterwards?
	Feelings then and now? Information and support you needed?
Now:	How do you feel about the whole experience now? Ongoing problems/ worries?
	Thinking about choice of place of birth? Feelings?
	Advice to others? Plans for next baby?
To close:	Anything that could have been done to make whole experience better?
	Anything to say to midwives? Suggestions for improvements?

### Analysis

Analysis began and continued during data collection so that emerging themes could be explored in later interviews. Data were coded systematically and analysed thematically using constant comparison and exploration of deviant cases [25]. The coding framework incorporated both themes anticipated from existing research, such as ‘hopes and expectations’, and emergent themes arising as analysis continued, such as ‘passivity’. Table 2 shows examples of codes and themes. Coding reports produced were subjected to further analysis, exploring areas of commonality between participants with shared characteristics and other patterns in the data [23]. In addition, large matrices or tables were produced in which each row represented one participant and each column represented a category or theme, similar to the charting stage of the framework approach [26]. These were used to summarise the data and to explore patterns in the data, for example, where similar or contrasting experiences were described by women transferred from the same type of midwifery unit or in similar circumstances e.g. in the context of prolonged labour. One author (RER) analysed the data; RF and JJK read a sample of ten transcripts and RF, JJK and LL discussed coding decisions, analysis and interpretation of the data with RER throughout. In the results which follow, extracts from women’s narratives are identified by pseudonyms along with an F for freestanding midwifery unit or an A for alongside midwifery unit to indicate the type of unit women were transferred from. Extracts have been edited for readability, for example removing repeated words and ‘ums’ and ‘ers’.

**Table 2:** Examples of codes and how these related to themes

Hopes and expectations	Choosing midwifery unit	Physical environment
		Social environment
		Friends’ / family experience
		No choice / hospital policy
	Hopes for birth	Ideal birth
		Normal / natural birth
		Birth plan
		Pain relief
	Expectations of transfer	Seeking information
		Seeking reassurance
		Blasé
In the midwifery unit	Midwifery care	Care and support
		Feeling understood
		Control
		Trust
	The decision to transfer	Preparation
		Feelings: disappointment
		Feelings: relief
		Feelings: anxiety, fear, worry
The transfer journey: in limbo	Change / contrast	Physical environment
		(Dis)comfort
		Social environment
		Others: midwife / ambulance staff/ husband
	Uncertainty / anxiety	Information
		Not knowing
		Fears and worries
		Journey time
	Passivity	Not asking
		Choices
Understanding why	Understanding what happened	Seeking information
		Finding resolution
		“Debrief”
		Feeling guilty
	Implications for the future	Plans for future babies
		Choices in future

## Results

Thirty women who had experienced transfer from a midwifery unit to an obstetric unit during labour or immediately after birth were interviewed. The first, pilot, interview took place in March 2009 with all others taking place between June 2009 and March 2010. Interviews were carried out by RER and lasted between 45 and 181 minutes (median 88 minutes).

### Characteristics of the sample

Women taking part were transferred from 21 different midwifery units, both freestanding and alongside, in many different parts of the country (Table 3). There was wide variation in the transfer experience of women. Transfers took place for a range of reasons including emergencies (intrapartum haemorrhage, hypertension, a concealed placental abruption and postpartum haemorrhage) and less urgent situations (failure to progress in the first or second stage of labour, meconium staining and fetal distress). Four women spent only a few minutes in a midwifery unit and were transferred in the early stages of labour; most women were transferred after a few or more hours of labour. Two women were transferred after having had their baby, both because of concerns about their own health. Transfer times varied from a few minutes to one hour. One third of the women gave birth vaginally without instrumental assistance, just less than one third with ventouse or forceps and just over one third had a caesarean section. In keeping with the population of women planning birth in freestanding midwifery units in England, but less so with the more diverse group planning birth in alongside units [1,6], the sample was predominantly White British, married or cohabiting and of relatively high socio-economic status. All but four women were transferred while having their first baby.

**Table 3:** Characteristics of participants

**Characteristic**	**No. of participants (n=30)**
**Location (Strategic Health Authority)**	
North West	4
Yorkshire and the Humber	1
West Midlands	3
East of England	1
London	4
South East Coast	3
South Central	10
South West	4
**Type of midwifery unit**	
Alongside midwifery unit	12
Freestanding midwifery unit	18
**Ethnicity**	
White British	26
White other	2
Asian	2
**Country of birth**	
UK	27
Non-UK	3
NS-SEC*	
1 Higher managerial & professional	8
2 Lower managerial & professional	19
3 Intermediate	1
4 Small employers and own account workers	1
5 Lower supervisory and technical	0
6 Semi-routine	0
7 Routine	1
8 Never worked and long-term unemployed	0
**Marital status**	
Married	19
Single (living with partner)	10
Single (living without partner)	1
**Age (years)**	
20-24	1
25-29	12
30-34	7
≥35	10
**Parity**	
Primiparous	26
Multiparous	4
**‘Type’ of transfer**	
Intrapartum emergency	5
Intrapartum non-emergency	18
Epidural request	5
Postpartum	2
**Type of birth**	
Non-instrumental vaginal	10
Ventouse	2
Forceps	6
Caesarean section	12
**Months between birth and interview**	
1-3	10
4-6	4
7-9	10
10-12	6

*National Statistics Socio-economic Classification.

### Findings

So yeah it's, it's not a small disappointment, it's a very big disappointment. It's absolutely enormous, you know… like somebody running a tractor through your wedding day.

Katelyn (F) used these striking words to sum up her feelings about her experience. Her words speak of hopes and expectations for a celebratory life event, and its associated memories, dashed and lost because of an unexpected catastrophic occurrence. Following the structure of women’s narratives, we describe women’s experience and feelings and explore how these were shaped by their expectations, environment, changing circumstances and by those around them.

### Hopes and expectations

Women commonly described going into labour wanting a “natural” birth, with the use of pain relieving drugs and medical intervention kept to a minimum. The degree of attachment to this ideal varied. A small number of women expressed the opinion that it is not possible to “plan” something as inherently unpredictable and uncontrollable as birth and to do so would risk disappointment if things did not go according to plan.

*… if you have a plan… and you've got that quite fixed in your head about how you want things to go and then it doesn't happen the way that you want it can be terribly distressing…* Adele (F)

Others said that to “try” for a natural birth was a sensible starting point; they wanted to avoid “unnecessary” medical intervention but would be “open” to intervention if required.

*I was quite happy for it to be as natural as possible to, to start with and then to, to see how it went.* Kimberley (A)

For most women a natural birth was their “ideal” birth, representing a more positive birth experience; women used the words “calm”, “stress-free”, “relaxed” and “enjoyable” to describe the birth they hoped for.

It was a commonly held view that the midwifery unit environment would enable and support this ideal birth and this was one reason why women chose to plan to have their baby in a midwifery unit. Charlotte (F), for example, like others, made an explicit link between the homely and relaxed environment which would enable her to be more relaxed and facilitate a normal labour:

… I’d be more relaxed and more capable of just sort of getting on with it…

Safiya (F) commented on the midwives’ explicit support for normal birth in the way that they worked:

All the midwives had nothing but supportive comment. They were very open and flexible; they were very pro sort of helping bring on natural labour, which is what we wanted…

Others talked of more pragmatic factors, such as car parking or proximity to home, influencing their decision-making, or were impressed by the recommendations of friends. A small number of women, all of whom were transferred from alongside units, did not make an active choice to have their baby in a midwifery unit, but did so simply because it was the hospital’s policy for women with apparently ‘straightforward’ pregnancies and early labour to be admitted to the midwifery unit. One woman in this situation was unaware that she was in a midwifery unit until after she had been transferred.

#### Expectations of transfer

Some women described thinking about the possibility of transfer during their pregnancy. A small number planned birth in an alongside unit to avoid the possibility of transfer in an ambulance from home or from a freestanding unit. Others described seeking out information about transfer rates or reassurance about the process of transfer:

*We asked about the percentage of women who were transferred out of the [midwifery unit] and were told that, and also asked sort of how long will it take the ambulance to arrive, how long will the journey be?* Carrie (F)

As with some other women who described this information as “hazy”, vague or imprecise, Charlotte (F) expressed some concerns about the information she and her husband were given:

Charlotte: *… but then when we went on this tour of the [midwifery unit]. Everybody asked the same questions about what are the statistics of people getting transferred to hospital and they said, “Oh we’ve only had four people transfer”.*

Intervewer: *Since?*

Charlotte: *Well exactly, and they’d opened last September… and they said they’d had like a hundred births there, so we were like, “Oh four out of a hundred, that’s not very many”. It’s only later we discovered they might have meant that month or something, I’m not entirely sure. In the end we couldn’t quite work out what statistics they were referring to.*

The most common comment from women on their thoughts about transfer during pregnancy, including from those who had sought out information, was that they just didn’t think it would happen to them. Women described themselves and their thinking as “blasé”, “idealistic”, “naïve” and “in denial”. A number, including Rose (F), reflected that while transfer was a theoretical possibility they either didn’t think it would happen or that it would be straightforward and therefore unproblematic:

I think I'd just got it into my head that it was really just going to be… a straightforward birth and also when we looked round [the hospital obstetric unit] they did say that if there was any problems it's only a quick journey down the motorway in an ambulance, it doesn't take long at all… under blue lights… so I thought, "Well if there is a problem I'll just get transferred really quickly…”

Some described this as a deliberate strategy; having positive thoughts about birth meant that they felt they were more likely to have a positive experience. Others described themselves as “hoping” they would be “lucky”, being “bloody-minded” or determined it wouldn’t happen to them, or that they “chose to blank out” the possibility of something happening during labour that would lead to transfer away from their chosen setting for birth:

*I think it was just something, because I didn't want it to happen… I kind of shut off to it.* Leanne (F)

*And… so I didn’t really give it any serious thought. I suppose also I didn’t really want to. [Laugh] … I suppose I possibly didn’t want to entertain that possibility, that it might not all go to plan.* Lily (A)

### In the midwifery unit

All the women in this study spent some time, from a few minutes to 12 hours, in a midwifery unit before transfer. Communication and interaction with the midwives looking after them featured heavily in women’s accounts. Most described broadly positive experiences in this regard, indicating that they felt cared for and supported by midwives. Hannah (F), for example, commented on the importance of the midwife acknowledging and involving her partner, even by a small gesture such as offering him tea and biscuits. Verbal and non-verbal encouragement through the pain of labour was appreciated. Katelyn (F) talked about how this helped her feel supported at a time when she felt vulnerable:

So… they just seemed personable and nice and had an easy smile and were quite happy to sort of touch your shoulder or give your hand a squeeze and some encouragement, and just that sort of confidence that everything’s going, you know, as it should, and it just helps you to relax, not worry that there’s something wrong…

Feeling that their midwives were aware of and understood their needs was also important. Carrie (F), for example, talked positively about her midwives understanding her needs in labour:

… they were very sensitive to where I was in my labour and what I was feeling and… I was very calm and self contained. I didn’t actually need to chat to anyone, I didn’t need anyone reassuring me, I just needed to focus and… obviously feel safe, but not to have someone constantly talking to me… So I think she, and then subsequently her colleague… were very sensitive to that. I think the first woman actually said, “Look, your husband’s in the room, I’m just going to go away . If you need me, call me, but you’re doing very well, I don’t see why you need me here.” And that was perfect for me, knowing that someone was sort of milling around outside the door.

In contrast, Olivia (A) described a long labour during which she came to feel “distressed”, “desperate” and that she was “going to die”, unable to communicate to her midwife how much pain she was in and that she needed help:

… nothing was happening and it wasn’t working and… throughout those two or three hours, I kept saying, “I can’t do it anymore and I need an epidural. It’s too much, it’s too painful…” And really it was just horrendous and the midwife kept saying, “Oh no, yes you can, yes you can, you can do it, you can do it,”… and I didn’t really know how to tell them that I can’t do it, how to… tell them that it is really, really, really terrible.

Olivia came to feel that her midwife did not understand how she was feeling or what she needed.

#### The decision to transfer

For some women, transfer was a response to an unequivocal emergency, but for most it was a ‘judgement call’ for the midwife, involving the weighing up of several factors including the woman’s and the baby’s immediate situation and, in some instances, the views of the woman herself. While the circumstances varied, there was some commonality in women’s responses to the decision to transfer and their feelings around that time.

Women commonly reported feeling “disappointed” at the decision to transfer. Disappointment was often about the loss or, as one woman said, the “disruption of the vision” of her ideal birth:

*So yes, there was a feeling of disappointment, that it wasn’t what I’d wanted, or wasn’t how I’d pictured it or imagined it.* Charlotte (F)

For Charlotte and for others, disappointment was also about not doing as ‘well’ as she had hoped, a sense of personal loss or even failure, of letting oneself down:

*… to be told you’re doing brilliantly, or to be told all the way through this is so efficient, and then to discover that actually you’re not so efficient at the second stage… When you’re sort of thinking, “For the first stage I sort of got ten out of ten from the teacher and now I’m not getting very much,”… it is a bit of a disappointment.* Charlotte (F)

Rose (F): *I think I was disappointed that I wasn’t having the birth that I wanted, or planned. …And I think I was a bit disappointed… in myself in a way for not…*

Interviewer: *Yeah, you even said cross with yourself didn’t you?*

Rose: *Yeah, just for not having sort of got on with it, if you know what I mean. [Laugh] There was nothing I could have done but… yeah, so I think that’s what I mean by disappointed.*

Disappointment for some women was also tinged with relief, as Leanne (F) described:

… because it wasn’t what we’d planned I was very disappointed, and I was also very tired by then, hadn’t slept for a night and we’d been up very early the morning before and… so it was a kind of relief that we were going to go somewhere and it was going to be hurried along, but also very disappointed.

Women who did not talk about feeling disappointed at the decision to transfer were transferred in an emergency (or in a situation that felt like an emergency) or, like Kimberley (A), had themselves requested transfer. Kimberley explained that she did not feel disappointed because transfer was a decision she had made herself:

Yeah, absolutely… it's why I feel positive about the experience, because it happened how I wanted it to at the time.

A small number of women described an overwhelming feeling of “relief” when they were first told of the need to transfer. Their accounts describe a negative or deteriorating experience in the midwifery unit, often in the context of prolonged labour, in which the time leading up to the decision to transfer was dominated by pain, exhaustion and feeling unable to cope. Zara (F) described feeling “distressed, frustrated and tired” as a midwife ‘encouraged’ her to try different positions in a long second stage. Having already discussed her preference to be transferred “earlier rather than later,” she described her feelings when the decision to transfer was made:

… and I actually thought, when I first bled and she said, “Right, we're going to move you”, I just had a wash of relief that, “Thank God,” because, “I can’t cope with that much”… it was a relief that I was going to be transferred.

Tamsin (F), who had experienced twelve hours in the first stage of labour in the midwifery unit with little ‘progress’, described her feelings when she was told that she would need to be transferred to the hospital:

How I felt was, “Yeah, I want to be there now,” you know? I was like literally, “Thank God.”…“Finally, finally, get me out of here.” I wanted to be out of that whole place. And really… from going in there and feeling, “Ahh, this is everything I want,” it became this… hellhole that I couldn’t stand to be around anymore… it was just… this vile, vile, awful place that I, I wanted to be away from.

This extract from Tamsin’s account is a vivid description of how she had come to feel about the midwifery unit by the time the decision to transfer was made. She, Zara (F) and some others described the decision to transfer as coming too late.

*I think they left it a bit late to transfer me personally. Because they waited., they didn’t move me whilst there was still an element of control, they called time when they really just couldn’t cope with it any more… and it made it into a traumatic affair…* Zara (F)

In contrast, for most women the timing of transfer was about right; while they felt disappointed, they were accepting of, or resigned to, the need to transfer. Some, like Charlotte (F) and Rachel (F), ascribed this to the trusting relationship that had been built up with their midwives and the degree of preparation for the idea that transfer might be required:

*And the encouragement I got… and just the general care from them… I trusted them, …both [name of husband] and I felt very comfortable with what we were being told at whatever stage, and we had sort of built up that level of trust that we didn’t have a problem with what they were saying.* Charlotte (F)

Rachel (F), who experienced persistent vomiting throughout her labour and was transferred when her labour slowed during the first stage, described her midwives leading her up to the decision to transfer by giving clear explanations about her progress, the results of urine tests and the possibility of needing to transfer. She explained that this helped her feel more comfortable with the decision to transfer and less anxious:

And as I say they did lead me up to it, it wasn’t a kind of complete shock, they gave me enough time to prepare myself and [for] it to be a calm experience as opposed to a panic. … So that, you know, so that my decision wasn’t needing to be [fingers snapping] made on the spot, it was a decision that was a progression and a process…

Women who experienced transfer in emergency situations understood the need for staff to act quickly, but also appreciated clear explanations and information. Kirsten (A) who was transferred after a sudden haemorrhage found the level of activity, coupled with bland reassurance, “isolating” and frightening. She explained the impact of a midwife saying to her, “You’ll be fine, your baby is fine”:

… and when I’m saying “What's happening, what's happening, what's happening to the baby?” and people are saying “It's fine, it's fine, it's fine,” that actually serves to make me more anxious 'cause I think, “They don't want to tell me what's going on.”

### The transfer journey

For women transferred from an alongside unit, the journey from one part of the hospital to another was largely uneventful, “quick and easy”. The exceptions to this were for women who were transferred in an emergency, for whom the sudden change of pace and environment could be frightening.

For women transferred from freestanding units an ambulance journey meant a move to a temporary, uncomfortable environment, which they described as “foreign” or “limbo”, for which they were largely unprepared. Rachel (F) described this stark contrast to the warm, comfortable and supportive environment that she had experienced in the midwifery unit.

The literal getting on the trolley and being pushed out into the ambulance. That bit as part of the transfer is really strange, ’cause you’re having to really transition from a lovely warm birthing pool, lovely room, calm music, to sound of a rackety metal trolley with a blanket over you, going into the cold, being put in the back of an ambulance. That’s quite a shock actually.

Others talked about an apparent loss of respect and dignity, which was again a contrast with their experience thus far. Leanne (F), like some other women, used humour to talk about this:

… strapped to an ambulance in [laugh] dodgy nightshirt and paper knickers, I was like, “No, please, I've never been out like this in my life.” [laugh]

Nevertheless the need to retain some sense of “decency” when moving from the warm and intimate environment of the midwifery unit was an important one. Leila (F) talked more explicitly about her concerns:

I was completely naked, you know, I’d come out of the water and I was in front of the midwives and they were all fine… Then my husband put my t-shirt, his t-shirt on me… I didn’t put any pants or anything on, just the t-shirt… Then I still had to get on to the trolley, and they had to put the blanket over me and put it up, ’cause it was January so it was cold outside but… that actually did make me feel a bit uncomfortable because I still felt exposed, you know, with the paramedics there, there’s two chaps… You feel a little bit violated, if you know what I’m saying.

#### Uncertainty and anxiety

Most of the women had never been in an ambulance before, were encountering ambulance staff for the first time, were travelling to a location that they knew little about and did not know what the outcome would be. The introduction of ambulance staff to the small group of people who had been looking after women during labour brought a change of emphasis that could be anxiety provoking, as Leanne (F) explained:

t [the midwifery unit] it was just so calm and relaxing and although they were saying, “Think you’re going to need a bit of help,” and it was like, “Oh okay, fine,” … suddenly it felt like doors flew open and these people came in and scooped me up and put me on a trolley and wheeled me out… it did feel like it was very rushed and very, “Quick, let’s go,” … which wasn’t ideal for me…

In contrast, where women described a negative or deteriorating experience in the midwifery unit, the changes associated with transfer could be seen as signs of hope or a new beginning and the introduction of different people could be a positive change. Hannah (F), who transferred without a midwife accompanying her, described feeling “calm” and “more relaxed” in the ambulance than she had been in the midwifery unit. Compared with the midwives, who she had found “cold” and unsupportive, the ambulance staff treated her with “care” and “compassion”. Tamsin (F) also described the change of personnel as reassuring:

I just remember feeling reassured by the ambulance staff… I remember being called sweetheart, you know, as they were getting me in there and it was just nice to have other people other than the midwives, and… it just felt like, “Oh there’s all these people that are taking me to somewhere where I can…” you know, they were just all part of… getting away from the birthing unit and getting away from the pain…

For most women, however, the changes associated with transfer were unsettling. Women described having fears about their babies’ or their own wellbeing, worries about the whereabouts and welfare of their husband or partner, concerns about the journey time and anxiety about what would happen at the hospital. Almost universally, women kept these worries to themselves and their unspoken questions were rarely anticipated or answered.

*I was fine. I felt quite… well I suppose withdrawn… I didn't talk a lot on there and I… just rolled over onto my side and had a bit of gas and air. I suppose I was… contemplating things actually. Thinking, “What am I going to expect this end? In [the hospital]?” I remember… I didn’t say that much to anyone on there.* Cheryl (F)

*… with every contraction I was just losing… blood so I was fearing … that (a) the baby would die … and also just fearful that I’d lost so much blood that actually I might have to lose my womb… because , you know, it was a long time and… you just know it’s definitely not right. So… I was just sort of… sobbing just quietly to myself, whilst lying on my side just… sort of fingers crossed.* Zara (F)

Rose (F) who experienced an hour long journey in an ambulance after more than 24 hours in labour described “dwelling on” how she would manage labour at the hospital in her “exhausted” state and whether she would need augmentation or a caesarean:

I should have asked somebody about that but I didn’t ask, I… was just thinking about loads of things, I think.

Thoughts and uncertainty about where they were going, what would happen when they arrived and who would be looking after them in the hospital featured in several accounts. Leona (F) was transferred during her second labour, to the same hospital where she had her first baby, and her midwife followed the ambulance in her car. Leona described not knowing where she was going, whether her midwife would continue to look after her and whether the hospital knew that she was coming, and went on to say why she would have liked to know these things:

I suppose for me it was to know that I wasn't going to be stuck in an ambulance giving birth or that they were prepared for me coming ‘cause…it sounds silly but you see on the programmes that… they might not have room and they might then turn you away and… just to be reassured that actually they are ready, when I get there it's all set up… and I could just do whatever I have to do… rather than worry about there being space.

Women also worried about the whereabouts and welfare of their husband or partner and valued the option to have their husband or partner with them in the ambulance. While for some women it was more practical to have their husband or partner follow the ambulance by car, most would have preferred to have the option of their husband or partner being able to travel with them and some, like Eileen (F), were distressed to find that they could not:

… when I got in the ambulance and I was having these contractions and they were hurting a lot more because I was laying down… and I wanted to be stood up… and then I just thought… “[Husband’s name] not here, and I want him here,”… and that’s when it was too much. [Laugh] So yeah… it would have definitely helped… ’cause I… wasn’t under any illusion that it wasn’t going to be painful, but you can’t really deal with the pain when you’re worried that your husband’s not going to make it to the birth. It made it lots, lots worse.

Journey time was also commonly mentioned by women transferred from freestanding units. Some women, like Rhiannon (A), described explicitly choosing to have their baby in an alongside midwifery unit because of concerns about transfer time:

… I thought, “Well that’s alright, I’ve only got to get in a wheelchair, up in the lift, and I’m there,” and it was the fact that it is… twenty minutes… in the ambulance, you know, and if it’s a serious problem… twenty minutes is a long time… before you… get to see a doctor.

Women transferred in an ambulance used language such as “fortunately it only took ten minutes” and references to the time of day and the amount of traffic also commonly featured:

*… and I was actually at the [hospital obstetric unit] probably within fifteen, twenty minutes. So the actual transfer bit, because it was a Sunday evening as well… one o'clock in the morning, it was quiet on the roads so the actual bit then was very good.* Diane (F)

Where journey times were longer, and particularly longer than the woman expected, this could be more difficult to cope with. Some women, like Leona (F), had anticipated that the journey in an ambulance would be at high speed and therefore faster than a car:

What I thought was going to be like a ten, fifteen minute rush job to the… [hospital obstetric unit] actually took 45 minutes because they went so slowly… which was absolutely horrendous at the time… because I wanted to push and… I was thinking, “We’ll be there in a minute, we’ll be there in a minute,” and it was just they were going very, very slowly… and it was a very long journey.

Factors such as these were beyond women’s control, often unanticipated, and could impact on women’s experience.

#### Passivity

Women’s accounts of the transfer journey reveal women as passive participants, in contrast with the more active role that many described in their experience of the midwifery unit. Being required to lie, strapped down, in the ambulance was an example of this, as Leanne (F) described:

I don’t think he [the ambulance staff] understood how uncomfortable I was on the stretcher and kind of not being able to move, because I’d been so active throughout the labour. …Even if I could have sat up in the ambulance [it] would have made a difference I think, but I wasn’t… given an option, it was just like, “Here’s the bed, are you on it? Strap you on, off we go.”… And I think maybe if I’d thought about it more and hadn’t been… in such a place in my head, I would have said… “Look, can I sit up? Can I move around, can I do this and whatever?”… but… I just felt that was a bit taken out… of my control because … I just was uncomfortable and didn’t feel like I could ask to be different.

A small number of women had the option of walking to the ambulance or were given the option or asked to be able to sit upright in the ambulance. Charlotte (F), who also had to lie down once in the ambulance, described how she was given some choices when the ambulance arrived, which she appreciated:

… when they did turn up they said, you know, did I want to walk to the ambulance or… did I want to have a wheel chair and I was like, “Oh I'll walk, and move around a bit,” … silly little things like they were saying, you know, “Do you want to put your trousers back on again?”

Once at the hospital all women were transported either in a wheelchair or on a trolley; some women would have appreciated the option of being able to walk.

### Continuity and discontinuity

Transfer, almost inevitably, leads to some discontinuity. One obvious discontinuity came at the doors of the hospital obstetric unit when the woman’s care was handed over to someone else. Most women had given little thought to the possibility of transfer and even less to the transfer process; if they were not told in advance that they would have a change of midwife, this could be an unpleasant surprise as Leila (F) described:

… it wasn’t very nice because… she’d been there the whole time, you know, through the second stage and while I was trying to push… so that didn’t feel very nice… I sort of wondered why she wasn’t staying with me, whether it was maybe the end of her shift… or she had to get back, I suppose, to [the midwifery unit].

The way in which the handover of care from one midwife to another was managed was important to women’s sense of continuity. Rachel (F) described being met by a midwife who was expecting her and explained why it was important that Rachel was present when the handover of care to this new midwife took place:

I witnessed that… which was really good, so it wasn’t sort of whisper, whisper behind closed doors… and that was good, that was important, because that again kept… communication open and it helped me understand what’s happening and didn’t feel… what are they talking about? Which at that moment is quite key, I think, because you don’t really know what’s going on particularly, you know things are changing all the time… so that was… important.

For a very small number of women continuity between the two settings was maintained because their midwife continued to care for them after transfer. This helped them feel safe in the care of someone with whom they had formed a trusting relationship and meant they had one fixed point of reference and an advocate in a potentially rapidly changing situation.

In contrast, Karla (F) and Rose (F) both described hour long transfer journeys by ambulance followed by cursory handovers of care, one of which took place in the car park, where they felt that the focus of the midwife was on returning to the midwifery unit in the ambulance. Karla described this as “abandonment” and Rose (F) reflected on how this impacted on her experience:

It would have been better if there’d been… a better transfer, because the questions that they were asking… were things like “When did you go into labour? How long have you been in labour? What length are your contractions?” And these are all things that I’ve been through so many times… with the midwives at [the midwifery unit]… I think the midwives from [the midwifery unit] could have answered all those questions… ’cause I really was struggling.

### Understanding why

The need to come to an understanding of “why” in relation to the events and circumstances leading up to and after transfer was commonly expressed in women’s accounts. Almost all would have appreciated an informal “debrief” conversation with a midwife or doctor with a view to ‘making sense’ of their experience, bringing “closure” and understanding whether there were any implications for future pregnancies.

A small number of women said that they did not feel the need to talk about their experience, while others sought this out and arranged it themselves, or were offered the opportunity directly. The comments of Rania (A) were typical of women who were not offered or had not yet had the opportunity to talk about their experience with a midwife or doctor:

… afterwards… it would have been nice to know in my particular instance… if there is a way of why things happen the way they did. … Maybe it’s just one of those things that you can’t explain… it happens and you just kind of have to deal with it, but it would be nice to… just have talked about my particular experience to someone…

Like Janet (A), some women did not actively ask questions and, in the immediate postnatal period, may not have been aware that they had any questions to ask, but would still have liked the opportunity to talk:

Janet (A): *Just to understand it a bit more really… 'cause it all goes in such a blur, 'cause you're scared and you're in pain… so it's all a bit sort of jumbled in your head really, so it would be nice just to get it set straight.*

Interviewer: *Mm. and did you have… unanswered questions or things that you wondered about particularly?*

Janet: *I don't think there was anything in particular. It just would have been nice to have sat with the people who… were there and gone through it.*

For women who described particularly traumatic labour and birth experiences, for the “debrief” conversation to be helpful, the timing, context and content was important. Abigail (A) described an attempted conversation on the postnatal ward, but said “I don’t think I took it in”:

There was… no moment at which I was sitting in a quiet room with somebody focused on me, you know, even for twenty minutes telling me what had happened without being interrupted.

The language used by women, getting it “set straight”, needing to “close that door”, “square it off”, “fill in the blanks” and “close the circle”, suggests that they felt that the opportunity to spend time with a midwife or doctor talking about their experience was or would have been helpful in ‘moving on’ from their experience, but many women did not have this opportunity and a small number who did, did not find the resolution they hoped for.

## Discussion

The women in this study went into their labour and birth experience expecting, or hoping for, a particular kind of birth and commonly this meant a “natural” birth. A small number of women had very strong feelings about their “ideal” birth. This included Katelyn (F), who described her experience as “a tractor through your wedding day” and contrasted it with her first birth which was as an “amazing experience” that “went exactly according to plan”. Most had less clear ideals and a few described themselves as “open” to or “expecting” intervention. Some had thought about transfer, but most just did not think that transfer would happen to them and looking back described themselves as “blasé” or “naïve”. Women’s expectations, their experience of labour and their perceptions of the way in which they were cared for, affected how women experienced transfer and how far it was perceived as a catastrophic event as in Katelyn’s case or as something that could be worked through and accepted.

Women were generally positive about their experience of care in the midwifery unit before transfer and described feeling supported by midwives who understood their needs. Most talked about feeling disappointed when the decision to transfer was made; disappointment was commonly about the loss of their anticipated “ideal” birth, while some also talked of a sense of personal loss or disappointment in themselves. Despite this disappointment, most were able to adjust to their changing circumstances and accept the need for transfer. Some ascribed this acceptance to the trusting relationship that had been built up with their midwives or the degree to which their midwives had prepared them for the idea that transfer might be needed. In contrast, a small number of women described a negative or deteriorating experience of their care in the midwifery unit, often, but not always, in the context of prolonged labour. They described feeling anxious, frightened, not in control and not safe, often perceived that they were transferred too late and were relieved to be transferred.

While a few women talked about the transfer journey in positive terms, women’s accounts commonly revealed the transfer journey as a period of ‘watchful waiting’ or ‘anxious anticipation’, a “limbo” period during which women wondered, worried or were fearful about what was to come and could be passive participants in a process which felt more like being “transported” than being cared for. This was a contrast with the care many had experienced in the midwifery unit. For women whose care was necessarily ‘fractured’ by transfer the best possible continuity was ensured by the same midwife continuing to care for the woman after transfer. Failing this, being met by hospital staff who appeared to care and a thorough handover of care in the presence of the woman maximised continuity of care. Finally, women expressed a clear need to understand and make sense of their experience and most appreciated or would have liked the opportunity to talk about their experience afterwards with a midwife or doctor.

The results of this study are consistent with other evidence from maternity care research. Women commonly described being given some information about transfer, knowing that transfer was a theoretical possibility, but at the same time not wanting to think about it or thinking that it would not happen to them. Qualitative research on women’s experience of operative delivery has revealed similar comments from women, for example, “I didn’t even read the chapter [on caesarean], it just wasn’t going to happen” [27]. The authors of this study suggested that this was indicative of a gap in antenatal education and information [27] and others have argued that antenatal care and education may not adequately prepare women for complications arising during labour [28,29]. In the study reported here, some women described the information they were given about transfer as vague or imprecise, so there may be room for improvement in terms of women’s preparation for transfer during pregnancy. However, it seems likely that women find it difficult to take in information about potential risks and complications during pregnancy. The authors of a meta-synthesis of research on women’s decision-making processes in relation to antenatal screening for Down’s syndrome describe women as “wishing to know and not wishing to know” about risks and possible outcomes, which has some resonance with the accounts of women interviewed for this study [30].

If women find it difficult to think about transfer during pregnancy, the support and preparation for transfer they receive during labour is likely to be even more important. A recent review of the literature on women’s experience of childbirth found that relationship with the caregiver, support and control were key themes emerging from the large body of research in this area [31]. While these are important factors in women’s perceptions of positive or negative experiences of care [32], they may be particularly important for women whose care is potentially fragmented through transfer. In this study, some women talked positively about being “prepared” for the need for transfer by their midwife and this appeared to help them adjust to their changing circumstances. Particularly negative experiences of care occurred often, but not always, in the context of prolonged labour and severe pain. Studies of women’s experience of prolonged and complicated labour have reported similar findings [33-36]. This suggests that the care of women with prolonged labour in midwifery units may need particular attention, with sensitive consideration by the midwife of when is the optimum time to transfer.

Other studies of women’s experience of transfer have not explicitly explored women’s experience of the ambulance or car journey from one birth setting to another [15-20]. Research on the journey from home to hospital in early labour for women in rural settings has also been carried out, but gives only limited insight into the experience of women who transfer, usually in established labour, from their planned birth setting to hospital [37,38]. While women’s experience is an explicit focus of midwifery care, women’s accounts suggest that during an ambulance journey, their experience was no longer an important focus; women felt less ‘cared for’ and more like being ‘transported’ at this time. The transfer journey is therefore an important and neglected area, where there is the potential to improve women’s experience. In this context it may be helpful to think of the ambulance as a ‘liminal space’, described by Horvath et al. as “in-between situations and conditions… characterized by the dislocation of established structures, the reversal of hierarchies, and uncertainty regarding the continuity of tradition and future outcomes” [39]. Women being transferred by ambulance are in effect in limbo between hospitals and between midwives; between natural birth and a more medicalised approach; potentially separated from their birth partner; and uncertain of the outcome. As anthropologist Victor Turner puts it, they are “neither here nor there; they are betwixt and between” [40]. Having choices, being supported by a husband or birth partner and being appropriately dressed or covered helped women feel cared for rather than transported and meant that the journey could be less distressing, but these choices were not always available to women. While the transfer journey could have been an opportunity for midwives to talk to women about their care and give them information about what to expect in the hospital obstetric unit, there was little evidence from women’s accounts of midwives using it in this way, leaving women with unanswered questions and concerns.

It is likely that the change in environment experienced and described by women during an ambulance transfer might also have an impact on midwives’ capacity to continue to provide high quality individualised care. However, there seems little rationale for the apparent variation in NHS policy on how many and which people are permitted to accompany a woman in an ambulance. The transfer journey is one area where some apparently straightforward changes to practice could make an important difference to women’s experience. Similarly, arrangements need to be in place so that midwives are not dependent on the returning ambulance for transport back to the midwifery unit and therefore have sufficient time to devote to a thorough handover of care.

Responsibility for the care of women who have been transferred should not end with the handover of care to hospital staff or when the woman is discharged home. Adjusting to a labour and birth experience that has not gone as expected is a process that extends into the postnatal period and beyond, even for women whose experience was not an overwhelmingly traumatic one. Women who have experienced transfer appreciate the opportunity to talk about their experience in order to understand and make sense of what happened and to help them plan for future pregnancies, but will not necessarily seek this out if it is not offered.

The most significant difference between the transfer experiences of women planning birth in the two types of midwifery unit was the transfer journey. This was a more important consideration for women transferred from freestanding units, although the brief comments of some women transferred from alongside units, particularly those transferred in an emergency situation, suggest that many of the same issues may apply. Beyond this, in terms of their experience of care before, during and after transfer, the accounts of women planning birth in the two types of midwifery unit revealed many common themes. Overall, in terms of implications for policy and practice, the findings of this study should be considered equally applicable to women planning birth in both types of midwifery unit.

### Limitations of the study

By using maximum variation sampling and a variety of recruitment approaches, this study aimed to represent as broad a range of experience as possible. As with other qualitative studies, the aim is not to obtain a statistically ‘representative’ sample or ‘generalisable’ results, but to represent a broad range of experience, including the ‘minority’ experience. In terms of socio-demographic characteristics, the sample interviewed for this study was less varied than was hoped, being predominantly White, older and more affluent than the national average. This reflects the characteristics of most women planning birth in most freestanding midwifery units, but does not reflect the wider social diversity seen in many alongside units [1,6]. It also proved difficult to identify and recruit women having a second or subsequent baby to take part in the study. This is in part because multiparous women are less likely to be transferred and therefore make up a minority of the population of women who have experienced transfer [1,6]. Finally, because all but two of the women interviewed were transferred during labour, before the birth of their baby, it was not possible to explore whether women who are transferred in the immediate postnatal period have particular concerns or support needs. Notwithstanding these issues there was wide variation across other important dimensions of experience. Women taking part in the study came from many different parts of England, had planned birth in both types of midwifery unit and had a wide range of different experiences and ‘stories’ to tell. There was no evidence that women who had more problems or who had a particular issue to raise were more motivated to come forward to take part in this study.

## Conclusions

Transfer affects a significant minority of women planning birth in midwifery units and is therefore a concern for women and their midwives. By exploring women’s experience of transfer and the impact of transfer on women, this study has provided evidence which can be used to improve women’s experience of midwifery unit care. Most women do not expect to be transferred and during pregnancy may find it difficult to take in information about the potential for complications to arise during labour. Supportive, sensitive care during labour, with preparation about the need for transfer, may help them to adjust to their changing circumstances. Particular sensitivity around decision-making may be required by midwives caring for women during prolonged labour. Midwives and ambulance staff have a role to play in ensuring that during the transfer journey women continue to feel cared for, supported and informed, and that they have the option for their husband or partner to accompany them. Where possible midwives should continue to care for women after transfer, but where this is not possible the woman and her partner should be made aware of this before arrival at the hospital obstetric unit and there should be a thorough verbal and written handover of care in the presence of or involving the woman and her partner on arrival. Women who have experienced transfer should be offered the opportunity to talk with a midwife from the midwifery unit about their experience and go through their maternity notes.

## Competing interests

The authors declare that they have no competing interests.

## Authors’ contributions

RER designed the study with advice from RF, JJK and LL. RER carried out interviews, analysed the data and drafted the manuscript. RF and JJK read a sample of interview transcripts and RF, JJK and LL discussed analysis and interpretation of data with RER throughout. RF, JJK and LL edited the manuscript. All authors read and approved the final manuscript.

## Pre-publication history

The pre-publication history for this paper can be accessed here:

http://www.biomedcentral.com/1471-2393/12/129/prepub
